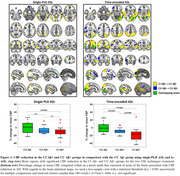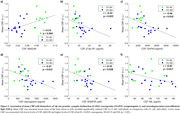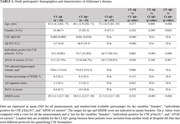# Unmasking early cerebral blood flow alterations with time‐encoded ASL in Alzheimer's continuum

**DOI:** 10.1002/alz.084419

**Published:** 2025-01-09

**Authors:** Carles Falcon, Paula Montesinos, Lena Vaclavu, Michalis Kassinopoulos, Carolina Minguillon, Karine Fauria, Diego Cascales‐Lahoz, José Contador, Aida Fernández‐Lebrero, Irene Navalpotro, Albert Puig‐Pijoan, Oriol Grau‐Rivera, Gwendlyn Kollmorgen, Clara Quijano‐Rubio, Jose Luis Molinuevo, Henrik Zetterberg, Kaj Blennow, Marc Suarez‐Calvet, Matthias J.P. Van Osch, Javier Sanchez‐Gonzalez, Juan Domingo Gispert

**Affiliations:** ^1^ Barcelonaβeta Brain Research Center (BBRC), Pasqual Maragall Foundation, Barcelona Spain; ^2^ Centro de Investigación Biomédica en Red Bioingeniería, Biomateriales y Nanomedicina (CIBER‐BBN), Instituto de Salud Carlos III, Madrid Spain; ^3^ Hospital del Mar Medical Research Institute (IMIM), Barcelona Spain; ^4^ Philips Healthcare Iberia, Madrid Spain; ^5^ Leiden University Medical Center, Leiden Netherlands; ^6^ Hospital del Mar Research Institute (IMIM), Barcelona Spain; ^7^ Barcelonaβeta Brain Research Center (BBRC), Barcelona Spain; ^8^ Centro de Investigación Biomédica en Red de Fragilidad y Envejecimiento Saludable (CIBERFES), Madrid Spain; ^9^ Servei de Neurologia, Hospital del Mar, Barcelona Spain; ^10^ Roche Diagnostics GmbH, Penzberg Germany; ^11^ Roche Diagnostics International Ltd., Rotkreuz Switzerland; ^12^ Department of Neurodegenerative Disease, UCL Queen Square Institute of Neurology, University College London, London, ‐ UK; ^13^ Wisconsin Alzheimer’s Disease Research Center, University of Wisconsin School of Medicine and Public Health, Madison, WI USA; ^14^ Hong Kong Center for Neurodegenerative Diseases, Clear Water Bay Hong Kong; ^15^ UK Dementia Research Institute at UCL, London UK; ^16^ Department of Psychiatry and Neurochemistry, Institute of Neuroscience and Physiology, the Sahlgrenska Academy at the University of Gothenburg, Mölndal Sweden; ^17^ Clinical Neurochemistry Laboratory, Sahlgrenska University Hospital, Mölndal Sweden; ^18^ Centro de Investigación Biomédica en Red de Fragilidad y Envejecimiento Saludable (CIBERFES), Instituto de Salud Carlos III, Madrid Spain; ^19^ Hospital del Mar Research Institute, Barcelona, Barcelona Spain

## Abstract

**Background:**

Arterial spin labelling (ASL) is a non‐invasive MRI technique for quantifying cerebral blood flow (CBF), used for monitoring changes over the course of a disease or treatment. A crucial parameter in ASL is the post‐labelling delay (PLD), determined by the time it takes for blood to travel from the labeling location to the tissue under investigation. Time‐encoded ASL (te‐ASL) utilizes multiple PLDs for more accurate quantification. This study aims to enhance our understanding of CBF changes across the AD continuum, emphasizing the utility of te‐ASL over single‐PLD ASL in detecting early CBF changes.

**Method:**

Fifty‐nine adults (≥ 60 years) along the AD continuum (24 cognitively unimpaired [CU] Aβ‐, 18 CU Aβ+, and 17 cognitively impaired [CI] Aβ+; Table 1) underwent CBF measurements using te‐ASL. Single‐PLD CBF measurements were derived based on the longest PLD (2000 ms) of the te‐ASL acquisition. CBF measurements were averaged across brain areas previously reported to be hypoperfused in AD. Associations between mean CBF and CSF biomarkers of Aβ, phosphorylated tau (pTau) proteins, synaptic dysfunction (GAP43, neurogranin, SNAP25, synaptotagmin‐1), and neurodegeneration (neurofilament light [NfL]), as well as cognitive scores, were investigated in CU participants. CSF Aβ42 and Aβ40 were assessed with Roche NeuroToolKit immunoassays, while pTau181 was measured with the Elecsys® Phospho‐Tau (181P) CSF immunoassay (Roche Diagnostics International Ltd). Sex and age were confounding variables.

**Result:**

te‐ASL exhibited superior sensitivity in detecting CBF hypoperfusion in CI Aβ+ subjects compared to single‐PLD ASL (Figure 1). Te‐ASL also revealed CBF hypoperfusion in CU Aβ+ subjects. In CU individuals, lower CBF correlated with decreased levels of CSF Aβ42/40 and increased levels of CSF pTau181, GAP43, neurogranin, SNAP25, and NfL (p < 0.05; Figure 2). No association was found between mean CBF and cognitive scores.

**Conclusion:**

This study provides compelling evidence that CBF reduction occurs earlier in the AD continuum than previously thought. te‐ASL emerges as a more sensitive tool than single‐PLD ASL for detecting subtle CBF changes across AD stages. Importantly, lower CBF in CU individuals correlates with multiple AD biomarkers, highlighting its potential as an early biomarker for AD progression.